# The impact of sunlight exposure on mortality of patients with end stage renal disease

**DOI:** 10.1038/s41598-019-38522-w

**Published:** 2019-02-18

**Authors:** Una Amelia Yoon, Yong Chul Kim, Hyewon Lee, Soie Kwon, Jung Nam An, Dong Ki Kim, Yon Su Kim, Chun Soo Lim, Jung Pyo Lee, Ho Kim

**Affiliations:** 1 Department of Biostatistics and Epidemiology, School of Public Health, Seoul National University, Seoul, Korea; 2 Department of Internal Medicine, Seoul National University Hospital, Seoul, Korea; 3 Department of Neuropsychiatry, Seoul National University Bundang Hospital, Seongnam, Korea; 4 Institute of Health and Environment, Seoul National University, Seoul, Korea; 5 Department of Internal Medicine, Seoul National University Boramae Medical Center, Seoul, Korea; 6 Department of Internal Medicine, Seoul National University College of Medicine, Seoul, Korea; 7 Kidney Research Institute, Seoul National University Hospital, Seoul, Korea; 8 Department of Medical Science, Seoul National University College of Medicine, Seoul, Korea

## Abstract

Recent data suggest that reduced sunlight exposure is associated with increased mortality in the general population. To date, the association between sunlight exposure and mortality in dialysis patients has not been examined. Among 134,478 dialysis patients in the Korean end-stage renal disease (ESRD) cohort from 2001 to 2014, 31,291 patients were enrolled from seven metropolitan cities, and data were analyzed using bi-directional case-crossover design. We examined the association between short-term sunlight exposure and mortality in ESRD patients. We adjusted for temperature, humidity, and daily concentrations of nitrogen dioxide (NO_2_), sulfur dioxide (SO_2_), ozone (O_3_), carbon monoxide (CO), and particle matter (PM_10_) as confounders. The characteristics of the study population included age (65.6 ± 12.26 (mean ± standard deviation [SD]) years), sex (male, 59.96%; female, 41.04%), comorbidity (diabetes, 53.58%; hypertension, 40.5%), and kidney dialysis type (hemodialysis, 73.02%; peritoneal dialysis, 26.98%). The mean ± SD follow-up time was 4.68 ± 4.37 years. The daily sunlight exposure was significantly decreased in the case group compared with the control group (P = 0.004). Sunlight exposure was associated with all-cause death overall (ORs [95% CI]: 0.99 [0.98–0.99], P = 0.042) in a fully adjusted model. Patients with diabetes (ORs [95% CI]: 0.98 [0.97–0.99], P = 0.016) or aged higher than 75 years (ORs [95% CI]; 0.97 [0.96–0.99], P = 0.020) had higher risks of mortality than patients without diabetes or aged below 75 years, respectively. These findings suggest that sunlight exposure is inversely correlated with all-cause mortality in dialysis patients.

## Introduction

There is a lively debate regarding the avoidance of sunlight and how this is a major risk factor for public health. Although high-intensity ultraviolet radiation can be a carcinogen to the skin^1^, there is growing scientific evidence that avoiding sunlight exposure is a major risk factor for various diseases and ultimately death^2–4^. Vitamin D is the “sunshine vitamin”, synthesized primarily in the skin during sunlight exposure, especially by ultraviolet B (UVB) radiation. In addition to observational studies, many randomized trials have demonstrated inverse associations of circulating vitamin D with the risks of cancer^5^, cardiovascular diseases^6–8^, infectious diseases^9^, metabolic disorders^10^, as well as mortality^11,12^. Prevalence of vitamin D deficiency is high in patients with chronic kidney disease (CKD) including end-stage renal disease (ESRD) patients undergoing dialysis^13,14^. In patients with CKD, conversion of serum 25-hydroxyvitamin D [25(OH)D] to 1,25(OH)_2_D, the active form of vitamin D, is insufficient owing to the loss of 1alpha-hydroxylase activity^15^.

Low circulating 25(OH)D concentration leads to mineral bone disease (MBD) such as secondary hyperparathyroidism, which is critically correlated with increased risks of coronary arterial calcification^16^, atherosclerosis, and endothelial cell dysfunction^17^, which result in cardiovascular events and mortality^18,19^. Although it is known that vitamin D supplementation improves serum 25(OH)D levels, there is still debate on whether this reduces mortality^20,21^.

Until recently, there have been few studies on the relationship between sunlight exposure and clinical prognosis in patients with ESRD who undergo renal replacement therapy, such as hemodialysis or peritoneal dialysis. Given this concern, this study was conducted to investigate the relationship of sunlight exposure and death in dialysis patients.

## Results

### Descriptive results

We identified 134,472 patients registered in the Korean Society of Nephrology for kidney dialysis registry. Among those, 31,291 (23.30%) patients died between 2001 and 2014 in the seven Korean metropolitan cities. The following characteristics of patients with all-cause deaths are shown in Table 1: sex (male, 59.96% and female, 41.04%), age at death (<75 years, 51.38%, and >75 years, 48.6%), comorbidity (diabetes, 53.58% and hypertension, 40.50%), kidney dialysis types (peritoneal dialysis, 26.98% and hemodialysis, 73.02%), primary disease (diabetes, 53.6%; hypertension, 15.2%; glomerulonephritis (GN), 8.16%; others, 7.31%; and unknown, 14.1%), and cause of death (cardiovascular disease, 28.31%; peripheral vessel disease, 12.7%; infection, 19.33%; cancer, 4.7%; and others, 34.9%). The average BMI in our study population was 21.21 for males and 21.20 for females. ESRD deaths occurred more frequently in males and those having diabetes as a primary disease. The number of ESRD deaths and the distributions by sunlight hours, temperature, humidity, and air pollutants (the daily concentrations of PM_10_, CO, NO_2_, SO_2_, and O_3_) concentrations by city in our study period are shown in Table 2.

**Table 1:** Baseline characteristics of the study population (2001–2014).

Variables	All-cause death
Overall	31,291
**Sex**	
Male (%)	18,448 (58.96)
Female (%)	12,843 (41.04)
Age (Death) (mean ± SD)	65.6 ± 12.26
<75 (%)	16,076 (51.38)
≥76 (%)	15,208 (48.6)
**Body mass index (BMI)**	
Male (mean ± SD)	21.21 ± 2.87
Female (mean ± SD)	21.20 ± 3.48
**Comorbidity**	
Diabetes (%)	53.58%
Hypertension (%)	40.5%
**Dialysis**	
Peritoneal dialysis (%)	8,442 (26.98)
Hemodialysis (%)	22,849 (73.02)
**Primary Disease**	
Diabetes	16,766 (53.6)
Hypertension	4,753 (15.2)
Glomerulonephritis	2,553 (8.16)
Others	2,287 (7.31)
Unknown	4,406 (14.1)
**Cause of death**	
CAD	8,859 (28.31)
PVD	3,976 (12.7)
Infection	6,049 (19.33)
Cancer	1,469 (4.7)
Others	10,928 (34.9)

Abbreviations: ESRD, end-stage renal disease; SD, standard deviation; BMI, body mass index; CAD, cardiovascular disease; PVD, peripheral vessel disease.

The mortality rate of this cohort was 23.3% among 134,472 ESRD patients.

**Table 2:** City-specific descriptive information on the study period, all-cause death, and the levels of environmental variables in ESRD patients (2001–2014).

City	Number of all-cause deaths	Number of monitoring sites	Sunlight (hrs.)		Temperature (°C)		Humidity (%)		PM_10_ (μg/m^3^)	
			Percentiles		Percentiles		Percentiles		Percentiles	
			50	90	50	90	50	90	50	90
Seoul	5,021	27	6.00	10.20	14.40	25.40	61.00	81.30	51.26	96.37
Busan	1,197	16	7.30	10.80	15.90	25.20	63.90	85.58	50.37	88.49
Daegu	856	11	7.20	10.80	15.65	26.80	57.90	79.28	50.18	88.49
Incheon	1,035	11	7.10	10.90	13.90	24.60	69.10	89.00	52.53	93.26
Gwangju	719	5	6.20	10.40	15.30	26.00	67.30	84.10	45.20	86.95
Daejeon	524	6	6.40	10.60	14.30	25.40	67.50	85.10	43.95	81.10
Ulsan	343	13	7.20	10.70	15.40	25.70	64.80	83.80	45.32	80.43
**City**	**Number of all-cause deaths**	**Number of monitoring sites**	**CO (ppm)**		**NO**_**2**_ **(ppm)**		**SO**_**2**_ **(ppm)**		**O**_**3**_ **(ppm)**	
			**Percentiles**		**Percentiles**		**Percentiles**		**Percentiles**	
			**50**	**90**	**50**	**90**	**50**	**90**	**50**	**90**
Seoul	5,021	27	0.607	1.002	0.037	0.056	0.005	0.008	0.053	0.087
Busan	1,197	16	0.473	0.786	0.024	0.037	0.006	0.009	0.056	0.087
Daegu	856	11	0.604	1.025	0.026	0.041	0.005	0.010	0.051	0.086
Incheon	1,035	11	0.596	0.958	0.028	0.046	0.007	0.011	0.055	0.089
Gwangju	719	5	0.597	0.976	0.023	0.035	0.004	0.006	0.048	0.075
Daejeon	524	6	0.588	1.096	0.021	0.035	0.004	0.008	0.018	0.035
Ulsan	343	13	0.479	0.772	0.020	0.031	0.006	0.010	0.024	0.037

Abbreviations: ESRD, end-stage renal disease; PM_10_, particulate matter less than 10 μm in diameter; CO, carbon monoxide; NO_2_, nitrogen dioxide; SO_2_, sulfur dioxide; O_3_, ozone.

### Effect modification by meteorological and air pollution factors

**Figure 1:**
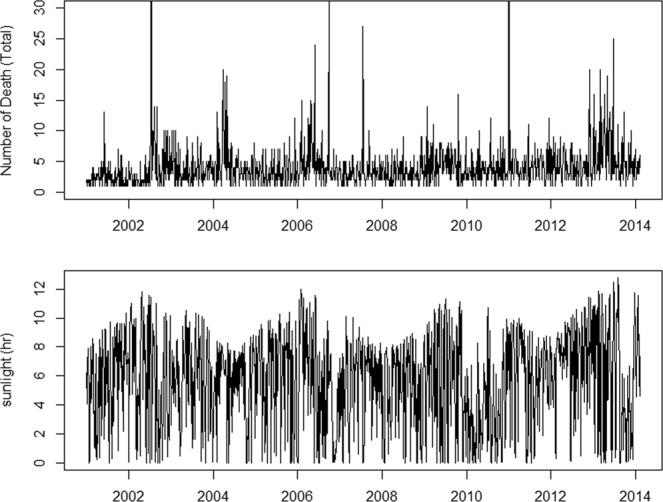
The trend of all-cause mortality and sunlight. The time-series plot indicates the daily number of mortality cases of ESRD patients from 2001 to 2014, (top panel) and daily hours of sunlight during 2001–2014 (bottom panel) which was collected from the Korea Meteorological Administration.

The distributions of the meteorological variables (daily sunlight hour, ambient temperature and humidity) and five major pollutants (the daily concentrations of PM_10_, CO, NO_2_, SO_2_, and O_3_) differed by city and are shown in Table 2. As shown in Supplementary Fig. S1, Temperature, humidity and O_3_ showed strong seasonal patterns. The time-series plots of both the number of deaths and exposure (sunlight hours) over time for the entire study period are shown in Fig. 1.

### Difference in daily levels of environmental variables between case and control periods (2001–2014)

Table 3 shows the daily levels of weather variables and the major five pollutants in the case and control periods. The difference in the daily level and our main exposure variable, sunlight hours per day, was significantly less in the case period compared to the control period, as shown by a t-test (p = 0.004).

**Table 3:** Difference in daily levels of environmental variables between case and control periods (2001–2014).

	Case Period		Control Period		Mean difference	95% CI	*P for t-test*
	Mean	SD	Mean	SD			
Sun light (hrs.)	5.638	3.906	5.777	3.935	−0.139	(−0.233, −0.046)	0.004
Temperature (°C)	13.813	10.148	13.824	10.191	−0.010	(−0.253, 0.233)	0.934
Humidity (%)	62.849	15.988	62.533	15.992	0.315	(−0.067, 0.698)	0.106
PM_10_ (μg/m^3^)	53.711	30.357	53.695	35.809	0.016	(−0.742, 0.774)	0.967
CO (ppm)	0.623	0.255	0.618	0.242	0.005	(−0.001, 0.011)	0.128
NO_2_ (ppm)	0.032	0.017	0.032	0.014	0.000	(0.032, 0.032)	0.600
SO_2_ (ppm)	0.006	0.002	0.006	0.002	0.000	(−0.00007, 0.00004)	0.652
O_3_ (ppm)	0.991	0.093	0.991	0.095	0.000	(−0.002, 0.003)	0.674

Abbreviations: SD, standard deviation; CI, confidence interval; PM_10_, particulate matter less than 10 μm in diameter; CO, carbon monoxide; NO_2_, nitrogen dioxide; SO_2_, sulfur dioxide; O_3_, ozone.

### Effect of sunlight exposure on ESRD death

**Figure 2:**
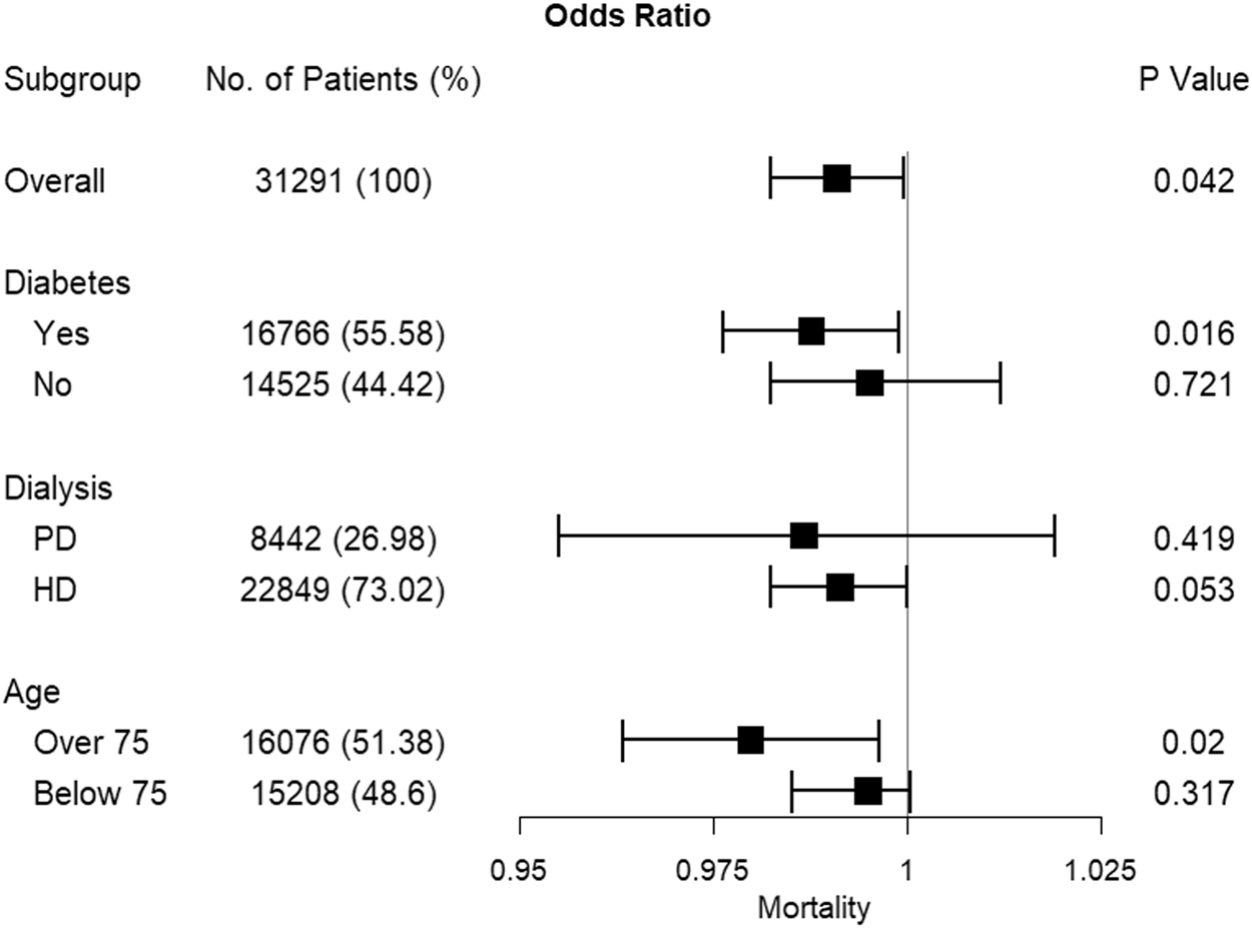
Percent decrease in odds ratios of sunlight exposures for all-cause death. Forest plot showing the associations of sunlight exposures and mortality of ESRD patients (adjusted for temperature, humidity and PM_10_), according to diabetes, dialysis modality, and old age (over 75 years). Abbreviations: ESRD, end-stage renal disease; PD, peritoneal dialysis; HD, hemodialysis.

**Figure 3:**
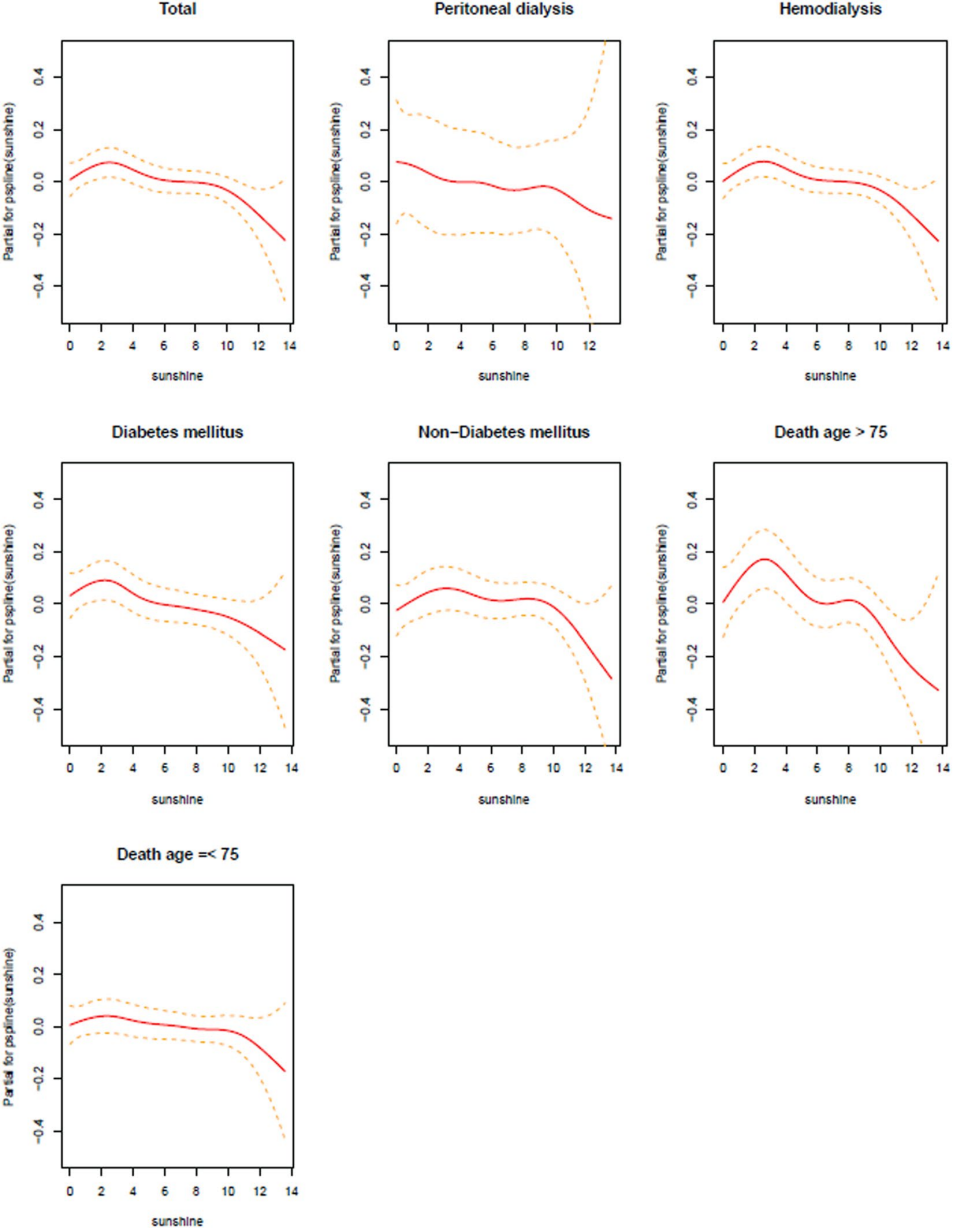
Penalized spline terms on exposure. An estimated exposure-response curve for short-term exposures to sunlight was created to assess the percentage increase in daily mortality at various daily sunlight exposure hours. The solid line represents the predicted log relative risk, and dashed lines indicate 95% confidence intervals.

There was a statistically significant negative association between daily sunlight hours and all-cause mortality in ESRD patients (Table 4 and Fig. 2). An increase in daily sunlight hours was associated with a decrease in the ESRD-related deaths (overall OR [95% CI]: 0.99 [0.98–0.99]). Moreover, in sub-group analysis we identified that patients with diabetes or being older than 75 years have higher mortality with lower sunlight exposure which means the risk of mortality was different across these groups (OR [95% CI] for diabetes: 0.98 [0.97–0.99], OR [95% CI] for being older than 75 years: 0.97 [0.96–0.99]). However, there was no difference of risk of all-cause mortality according to the dialysis types (OR [95% CI] for peritoneal dialysis: 0.98 [0.95–1.01], OR [95% CI] for hemodialysis: 0.99 [0.98–1.00]). Although patients receiving hemodialysis had a higher relative risk, as indicated in Table 4, it was not statistically significant (p = 0.054). These analyses were performed with PM_10_ as an air-pollutant potential confounder in Models 1 to 5, with a similar pattern in all models (Table 4 and Fig. 2). The penalized smoothing spline between sunlight exposure and ESRD death in Fig. 3 also illustrates that lower sunlight exposure was associated with increased risk of mortality.

**Table 4:** Effect modification of association between all-cause death and sunlight exposure with environmental confounders in conditional logistic regression models.

		Total		DM		Non-DM		PD		HD		Over 75		Below 75	
		*Beta*	*P*	*Beta*	*P*	*Beta*	*P*	*Beta*	*P*	*Beta*	*P*	*Beta*	*P*	*Beta*	*P*
Model 1	Sunlight (hrs.)	−0.009	0.042	−0.015	0.016	−0.002	0.721	−0.013	0.419	−0.009	0.053	−0.021	0.02	−0.005	0.317
	Temperature (°C)	0.001	0.781	0.001	0.888	0.002	0.788	0.013	0.351	−0.001	0.765	0.012	0.117	−0.004	0.411
	Humidity (%)	−0.001	0.667	−0.002	0.251	0.001	0.666	−0.003	0.561	0.000	0.796	−0.003	0.244	0.001	0.541
	PM_10_ (μg/m^3^)	0.000	0.838	0.0004	0.459	0.000	0.624	−0.001	0.675	0.000	0.889	0.0004	0.653	0.000	0.791
Model 2	Sunlight (hrs.)	−0.009	0.044	−0.015	0.018	−0.003	0.672	−0.015	0.359	−0.009	0.060	−0.021	0.017	−0.005	0.341
	Temperature (°C)	0.002	0.647	0.000	0.999	0.004	0.560	0.022	0.152	−0.002	0.617	0.014	0.077	−0.004	0.321
	Humidity (%)	−0.001	0.654	−0.002	0.309	0.001	0.609	−0.002	0.619	0.000	0.818	−0.003	0.277	0.001	0.560
	NO2 (ppm)	0.427	0.707	1.383	0.266	−2.463	0.263	−8.970	0.099	0.958	0.388	−2.848	0.311	1.059	0.369
Model 3	Sunlight (hrs.)	−0.009	0.039	−0.015	0.014	−0.002	0.717	−0.013	0.439	−0.009	0.048	−0.021	0.020	−0.005	0.303
	Temperature (°C)	0.002	0.647	0.002	0.755	0.002	0.733	0.010	0.478	0.000	0.945	0.012	0.104	−0.003	0.488
	Humidity (%)	−0.001	0.661	−0.002	0.314	0.001	0.646	−0.003	0.561	0.000	0.805	−0.003	0.003	0.001	0.553
	SO_2_ (ppm)	5.125	0.482	−2.914	0.769	−7.830	0.467	16.750	0.519	−7.269	0.326	−11.51	0.454	−2.924	0.717
Model 4	Sunlight (hrs.)	−0.009	0.041	−0.015	0.014	−0.002	0.748	−0.012	0.458	−0.009	1.504	−0.021	0.019	−0.006	0.237
	Temperature (°C)	0.001	0.719	0.001	0.875	0.002	0.726	0.017	0.243	−0.001	0.762	0.010	0.192	−0.003	0.434
	Humidity (%)	−0.001	0.641	−0.002	0.345	0.001	0.721	−0.004	0.459	0.001	0.707	−0.003	0.296	0.001	0.442
	O_3_ (ppm)	−0.178	0.791	0.434	0.633	−0.888	0.380	−3.987	0.136	1.504	0.428	1.086	0.457	3.110	0.146
Model 5	Sunlight (hrs.)	−0.009	0.046	−0.015	0.017	−0.002	0.739	−0.014	0.402	−0.009	0.060	−0.021	0.020	−0.005	0.352
	Temperature (°C)	0.000	0.966	−0.001	0.884	0.001	0.912	0.016	0.266	−0.003	0.388	0.013	0.097	−0.006	0.156
	Humidity (%)	−0.001	0.569	−0.002	0.251	0.001	0.671	−0.002	0.637	0.000	0.987	−0.003	0.296	0.001	0.721
	CO (ppm)	0.093	0.221	0.146	0.154	0.027	0.812	−0.268	0.361	0.158	0.033	−0.117	0.445	0.192	0.020

Abbreviations: DM, diabetes mellitus; PD, peritoneal dialysis; HD, hemodialysis; PM_10_, particulate matter less than 10 μm in diameter.

## Discussion

This is the first study, to our knowledge, to examine the association between short-term sunlight exposure and mortality, in a nationwide ESRD cohort in Korea using a bi-directional case-crossover method. In the present study, the daily sunlight exposure hours were measured in the seven metropolitan cities along with ambient temperature and relative humidity from 2001 to 2014 (14 years). The daily concentrations of PM_10_, NO_2_, SO_2_, O_3_ and CO were adjusted for as confounders. We have demonstrated that low-level daily sunlight exposures significantly increased the risk of all-cause mortality in dialysis patients, especially in high-risk patients with diabetes and old age >75 years.

To date, there has been limited research on the relationship between sunlight exposure and mortality in ESRD patients. In 2014, Shapiro *et al*. reported that among 47,286 US dialysis patients, those residing in higher ultraviolet (UV) index regions had lower all-cause mortality compared to those living in moderate-high UV regions^22^. The average of the annual noon-time UV index value was calculated in each patient during the follow-up periods. In our study, we applied a case crossover method and compared the daily sunlight exposure on the day of the mortality event with the daily sunlight exposure for 1 or 2 weeks on the same day of the week. Findings from our study are similar to Shapiro’s report that sunlight or UV exposure has a negative correlation with mortality in dialysis patients, but differed in that we also observed the effect of short-term sunlight exposure on mortality.

The principal response to sunlight exposure is elevation of vitamin D status. It is reported that the mean serum concentration of 25(OH)D was about 115 mmol/L in two traditionally living populations in East Africa with lifelong exposure to tropical sunlight^23^. In a cross-sectional study of patients with CKD inhabiting a subtropical region of Brazil where sunlight exposure is elevated throughout the year, the serum 25(OH)D levels were higher than those found in patients residing in higher-latitude regions^24^. Moreover, narrow-band UVB exposure is known to increase serum 25(OH)D and 1,25(OH)_2_D in dialysis patients^25^.

It is well known that vitamin D deficiency, which is caused by limited sunlight exposure, is highly prevalent in patients with ESRD and is associated with various adverse outcomes including death^14,26,27^. Cardiovascular disease is one of the most common cause of death in ESRD, which is in concordance with our data, while moderate to severe vitamin D deficiency is a risk factor for developing cardiovascular disease. Previous studies showed that a lower serum 25(OH)D concentration is associated with an increased risk of cardiovascular events, in not only peritoneal dialysis^28^, but also hemodialysis patients^26^.

Infectious disease remains the leading cause of death in ESRD patients. Increasing evidence demonstrates that vitamin D has immune-modulatory effects on both the innate and adaptive immune systems^29^. 1,25(OH)_2_D is known to suppress adaptive immunity by inhibiting the proliferation and differentiation of CD4 cells into T helper cell type 1 (Th1) and Th17 cells and by promoting the production of Th2 and regulatory T cells^30^. Macrophages activated by toll-like receptors (TLRs) promote the production of 1,25(OH)_2_D, which then induces the expression of the antimicrobial peptides, cathelicidin^31^. The association of vitamin D deficiency and infectious events, such as septic shock^32^, respiratory infection^33^, and influenza^34^, is supported by a large number of epidemiologic studies.

As reported previously, several observational studies have shown that vitamin D supplementation may be associated with a reduced risk for cardiovascular and all-cause death among ESRD patients^35–38^. Contrary to this, a recent review article with 17 randomized controlled trials (RCTs) involving 1,819 patients demonstrated that vitamin D treatment did not affect the risk of cardiovascular or all-cause death^20^. These differences between observational studies and RCTs might be explained by selection bias, because patients taking vitamin D supplements are generally healthier than untreated patients. Therefore, large-scale RCTs are needed to assess the efficacy of vitamin D treatment for ESRD patients.

Although there has been substantial body of evidence that sunlight exposure, through synthesis of vitamin D, might have a beneficial effects including survival gain in many kinds of diseases, controversies still exists with “vitamin D hypothesis”. In a large prospective study of Swedish women, natural sunlight exposure was inversely associated with all-cause mortality and cardiovascular mortality, whereas artificial UV exposure using indoor tanning devices^4^. Furthermore, in a recent study, overall cancer mortality was not associated with baseline 25(OH)D status in the general population of NHANES III cohort^39^. It is important to note that vitamin D might not be the only pathway whereby sunlight exposure, especially natural source of sunlight might have beneficial effects on human health. It has been reported that sunlight induces Nitric Oxide (NO) synthesis in the skin and it is released the systemic vasculature which acts as a vasodilator and lowers blood pressure^40^. Furthermore, UV radiation-induced NO showed suppressive effect in developing obesity and metabolic syndrome in a mouse model which of the two mechanism were independent of vitamin D supplementation^41^.

The strengths of our study include the examination of a large, contemporary Korean dialysis population with long-term follow-up of 14 years, and the comprehensive availability of clinical data allowing for adjustment of multiple weather and air-pollution confounders. Moreover, this study employed a case-crossover design, which is particularly powerful for matching potential confounding factors individually and avoiding the selection bias, healthy-day bias, and healthy-volunteer bias that are limitations of ecological epidemiologic studies^42^. Although sunlight exposure has seasonality, such as shorter sunlight hours during the winter period (December, January, and February) compared to other seasons, we did not need to adjust for the season or holiday as cofounders because we made the controls in the same day of the week within two weeks in the case-crossover design. However, several limitations of our study bear mention. First, we were unable to measure the serum vitamin D concentrations in our study population. Second, given that the Korean National Institute of Environmental Research measures the sunlight exposure time only in major cities, our study cohort may not be representative of dialysis patients living in outlying or rural regions.

For the selection of control days in the case-crossover design, several selection schemes have been used and compared. A recent study showed that the bi-directional and three different time-stratified (day of the week) methods yielded no difference in results in the assessment of the association between air pollution exposure and acute myocardial infarction^43^. This experimental comparison of the control selection schemes in the case-crossover study design could support the fact that our use of the bi-directional method in selecting control days would likely produce unbiased estimates.

In conclusion, in the Korean ESRD population from 2001 to 2014, sunlight exposure was inversely correlated with increased risk of all-cause mortality. Further studies are needed to evaluate the effect of extended sunlight exposure and vitamin D supplement on survival.

## Methods

### Study population

The Korean Society of Nephrology (KSN) end-stage renal disease (ESRD) registry was established in 1985. All KSN members contribute voluntarily to the ‘Insan Prof. Byung-Suk Min Memorial ESRD Patient Registry^44,45^. The KSN ESRD Registry Committee has been collecting data on dialysis through an online internet program that was opened in 2001 and revised in 2013. Informed written consent was obtained from all the patients who were enrolled in the registry. The Korean ESRD registry covers about two-thirds of all dialysis patients in Korea because the enrollment is voluntarily. The present study enrolled patients who died from 2001 through 2014, according to KSN data. The study protocol complies with the Declaration of Helsinki and received full approval from the institutional review board at the Seoul National University Boramae Medical Center (20180410/10-2018-50/051). Clinical information, including the date of death, age, sex, body mass index (BMI), comorbidities (hypertension and diabetes mellitus) and type of kidney dialysis (peritoneal dialysis and hemodialysis), the causes of ESRD, and the cause of death were obtained from the registry.

### Weather and air pollution data

The seven metropolitan cities in Korea were selected as our study area, and 2001 to 2014 was chosen as the study period. We obtained hourly data on ambient temperature, relative humidity, and hours of sunlight from the Korea Meteorological Administration. To adjust for potential confounding factors, we also obtained the daily (24-hour) concentrations of nitrogen dioxide (NO_2_), sulfur dioxide (SO_2_), ozone (O_3_), carbon monoxide (CO), and particulate matter less than 10 μm in diameter (PM_10_) measured at 89 monitoring sites located within the seven cities. These were provided by the Korean National Institute of Environmental Research. As short-term confounder measures, we used the daily max concentration of ozone (O_3_) and the daily mean concentration for the other air pollutants (PM_10_, NO_2_, SO_2_, O_3_ and CO) within same city.

### Study design

**Figure 4:**
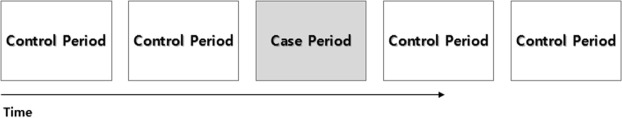
Bi-directional sampling of control time in the case-crossover study. A bi-directional case-crossover study was conducted, which sampled control periods as the exposure seven days before and seven days after the event day (the day of mortality), producing four control days per case.

We applied a bi-directional case-crossover design (Fig. 4) to estimate the short-term association between sunlight and ESRD death. A case-crossover design, which was described by Maclure for evaluating a transient acute effect, is a variant of the case-control design and produces sufficient statistical power with small cases^46^. It has recently been used as an alternative to time series, and an extension of this approach has also been used for observational studies in areas such as clinico-epidemiology, impairment epidemiology, pharmaco-epidemiology and environmental epidemiology. The case period was defined as the day ESRD led to the death of the patient. We performed the bi-directional case-crossover study as a two-to-one matched case-control study that sampled control periods as the exposure, seven days before and seven days after the date of event (ESRD death); resulting in four control days per case^47^ (Fig. 1). For example, if ESRD death occurred on April 13, the four control days were selected as follows: March 30, April 6, April 20, and April 27. In the case-crossover design, the time-invariant individual characteristics, such as sex and genetic predisposition, and the slowly varying characteristics, such as age, marital status, employment status, and seasonality, can be controlled^48^. In our study, the daily sunlight exposure hours during the case and control periods were compared.

### Statistical analysis

The conditional logistic regression analytic method is an extension of the logistic regression method, which allows one to take into account the stratification and matching that is usually utilized in observational studies. We investigated the association between sunlight and ESRD death risk by conditional logistic regression performed via the Cox proportional hazard function. The comparisons within subject were made between the case and control periods. The odds ratios (ORs) and 95% confidence intervals (CIs) for the risk of ESRD death on sunlight exposure were calculated by conditional logistic regression analysis. We used ambient temperature and humidity as potential confounders, and five other air pollutants (PM_10_, NO_2_, SO_2_, O_3_ and CO) were also used to have five different models as a potential effect modification. The conditional logistic model can be simplified by the following formula after matching for the time-invariant individual risk factors;

$$\mathrm{log}\,\frac{{{\rm{p}}}_{{\rm{ij}}}}{1-{{\rm{p}}}_{{\rm{ij}}}}={{\rm{\alpha }}}_{{\rm{i}}}+{{\rm{\beta }}}_{1}\times {{\rm{sunlight}}}_{{\rm{ij}}}+{{\rm{\beta }}}_{2}\times {{\rm{temperature}}}_{{\rm{ij}}}+{{\rm{\beta }}}_{3}\times {{\rm{humidity}}}_{{\rm{ij}}}+{{\rm{\beta }}}_{4}\times {\rm{air}}\,{{\rm{pollutants}}}_{{\rm{ij}}},$$

where β_1_, β_2_, β_3_ and β_4_ represent the vectors whose components denote the log odds of mortality associated with sunlight, ambient temperature, humidity, and air pollutants separately as confounders. Using the formula above, ${Y}_{ij}\,{\epsilon }\{0,1\}$ represents the case status (case = 1, control = 0) of the *j*th observation of *i*th strata where *α*_*i*_ is constant term of *i*th strata. The ambient temperature and humidity are stationary confounder factors and five air pollutants (PM_10_, NO_2_, SO_2_, O_3_ and CO) were applied in each model.

For subgroup analysis, we defined higher risk patients in the ESRD cohort as those with diabetes, older adults (over 75 years), or those undergoing peritoneal dialysis or hemodialysis, and we compared them to the lower-risk group. The statistical analysis was performed using the statistical programing language R, version 3.4.0.

## Supplementary information

Supplemental materials

## Data Availability

The datasets generated during and/or analyzed during the current study are available from the corresponding author on reasonable request.
