# Supplementary material for: The impact of sunlight exposure on mortality of patients with end stage renal disease
Source: Sci Rep. 2019 Feb 18;9:2230. doi: 10.1038/s41598-019-38522-w (PMC6379426; doi:10.1038/s41598-019-38522-w)
Supplement: Supplementary file 1 — Supplemental materials [file 41598_2019_38522_MOESM1_ESM.docx]

**The impact of sunlight exposure on mortality of patients with end stage renal disease**

Una Amelia Yoon^1^, Yong Chul Kim^2^, Hyewon Lee^3,4^, Soie Kwon^2^, Jung Nam An^5^, Dong Ki Kim^2,6^, Yon Su Kim^7,8^, Chun Soo Lim^5,6^, Jung Pyo Lee^5,6^, Ho Kim^1,4^

Una Amelia Yoon and Yong Chul Kim contributed equally to this work.

**Author Affiliations**

*^1^Department of Biostatistics and Epidemiology, School of Public Health, Seoul National University, Seoul, Korea*

*^2^Department of Internal Medicine, Seoul National University Hospital, Seoul, Korea*

*^3^Department of Neuropsychiatry, Seoul National University Bundang Hospital, Seongnam, Korea*

*^4^Institute of Health and Environment, Seoul National University, Seoul, Korea*

*^5^Department of Internal Medicine, Seoul National University Boramae Medical Center, Seoul, Korea*

*^6^Department of Internal Medicine, Seoul National University College of Medicine, Seoul, Korea*

*^7^Kidney Research Institute, Seoul National University Hospital, Seoul, Korea*

*^8^Department of Medical Science, Seoul National University College of Medicine, Seoul, Korea*

**Corresponding authors:**

Jung Pyo Lee, MD, PhD

Associate Professor

Department of Internal Medicine, Seoul National University Boramae Medical Center and Seoul National University College of Medicine

20 Boramae-ro 5-gil, Dongjak-gu, Seoul 07061, Republic of Korea.

E-mail: [nephrolee@gmail.com](mailto:nephrolee@gmail.com)

and

Ho Kim, PhD

Professor

Department of Biostatistics and Epimediology, School of Public Health, Seoul National University and Institute of Health and Environment, Seoul National University

Room 708, Building 220, Graduate School of Public Health, Seoul National University, 1 Gwanak-Ro Gwanak-Gu, Seoul 08826, Korea

E-mail: [hokim@snu.ac.kr](mailto:hokim@snu.ac.kr)

**Supplemental Figure 1: Daily weather and five major air pollutants from 2001 to 2014.**

The time-series plot indicates the seasonal distribution of the meteorological variables (daily sunlight hour, ambient temperature and humidity) and five major pollutants (the daily concentrations of PM10, CO, NO2, SO2, and O3) from 2001 to 2014.

**
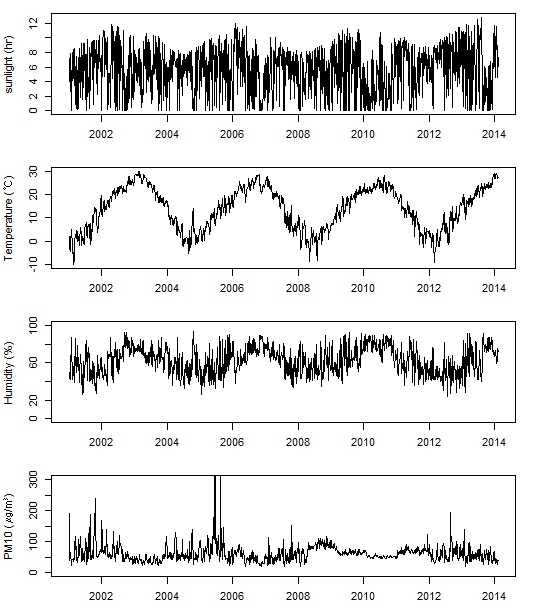

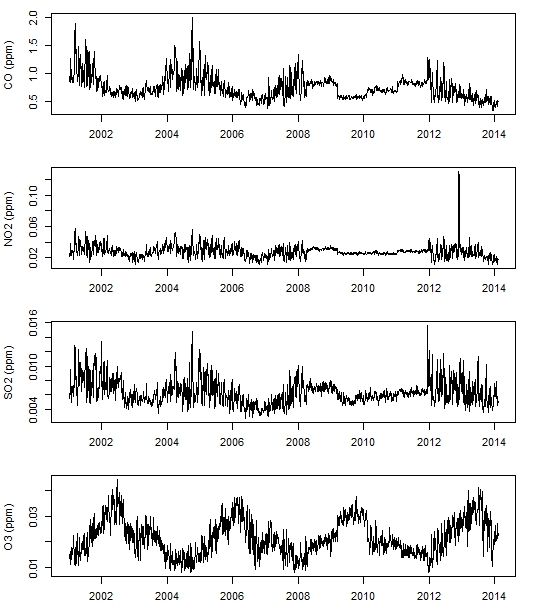
**
